# The role of homologous recombination in human retrovirus-associated diseases

**DOI:** 10.1007/s11262-025-02210-x

**Published:** 2025-12-20

**Authors:** Mohammad Mehdi Akbarin, Zahra Farjami, Gabriel Eduardo Acevedo-Jiménez, Cecilia Rodríguez Murillo, Víctor David González-Fernández, Lucero de María Ávila-De la Vega, Hugo Ramírez Álvarez

**Affiliations:** 1 Virology, Genetics, and Molecular Biology Laboratory, Faculty of Higher Studies Cuautitlan, National Autonomous University of Mexico, Veterinary Medicine, Campus 4, Cuautitlan Izcalli, Mexico; 2 Mashhad Medical Sciences-Medical School, Islamic Azad University, Mashhad, Iran; 3 Department of Biology, Damghan Branch, Islamic Azad University, Damghan, Iran; 4 Universidad Nacional Autónoma de México. FES-Cuautitlán, Cuautitlán-Teoloyucan Highway, Km. 2.5, San Sebastián Xhala, Zip Code 54714 Cuautitlán Izcalli, State of Mexico Mexico

**Keywords:** Retrovirus, HIV, HTLV, Homologous recombination, DNA repair, RAD51

## Abstract

Human retroviruses such as HIV-1 and HTLV-1 hijack host cellular mechanisms for their replication, survival, and pathogenesis, often causing profound genomic instability. This review explores the dual role of homologous recombination (HR), explicitly mediated by the recombinase RAD51, in the context of retroviral infections. RAD51 is central to high-fidelity repair of DNA double-strand breaks, yet its activity is manipulated differently by HIV-1 and HTLV-1. In HIV-1 infection, RAD51 expression is elevated by viral proteins like Tat and Vpr, promoting DNA repair and enhancing viral transcription through interactions with NF-κB, thereby supporting viral persistence. Conversely, HTLV-1 suppresses RAD51-mediated HR via viral proteins such as p30 and Tax, promoting error-prone DNA repair pathways that contribute to oncogenesis. These contrasting effects may underscore RAD51's functional plasticity as both a facilitator of viral replication and a potential antiviral restriction factor. Furthermore, the therapeutic modulation of RAD51 activity-especially in combination with PARP inhibitors offers promising avenues for treating retrovirus-associated malignancies such as adult T-cell leukemia/lymphoma. This review highlights RAD51 as a pivotal connection in the interplay between genome stability and retroviral pathobiology.

## Introduction

Human retroviruses, particularly Human Immunodeficiency Virus (HIV) and Human T-cell Leukemia Virus (HTLV), are responsible for a broad range of diseases, including acquired immunodeficiency, neurodegeneration, and malignancies [1, 2]. These viruses share a unique replication strategy involving the reverse transcription of their RNA genome into DNA, followed by integration into the host genome [1]. This integration is essential for viral persistence but can significantly disrupt host cellular homeostasis. The random or semi-targeted insertion of proviral DNA can lead to insertional mutagenesis, dysregulation of gene expression, and genomic instability, setting the stage for cellular transformation and oncogenesis [1]. For example, HTLV-1 is the established cause of adult T-cell leukemia/lymphoma (ATLL). At the same time, HIV-1 infection increases the risk of various cancers, either directly through viral effects or indirectly through long-term immune suppression and chronic inflammation [3–5].

One of the most serious consequences of retroviral integration is the induction of DNA double-strand breaks (DSBs), which can destabilize the genome if not correctly repaired [6, 7]. Cells have evolved a sophisticated set of mechanisms to detect and repair such DNA lesions, among which homologous recombination (HR) stands out as a high-fidelity repair pathway [6, 8]. HR plays a crucial role in the accurate repair of DSBs by using the undamaged sister chromatid as a template. This pathway is tightly regulated and primarily active during the S and G2 phases of the cell-cycle [9, 10]. A growing body of evidence suggests that retroviruses may both induce DNA damage and exploit the host's HR machinery to facilitate aspects of their life cycle or to ensure the survival of infected cells [11, 12]. As such, understanding how retroviral infections interact with HR-mediated repair pathways provides critical insights into viral pathogenesis and the emergence of virus-associated malignancies.

At the core of the HR pathway is RAD51, a key recombinase responsible for homologous strand pairing and exchange [13]. RAD51 is indispensable for the search and invasion of homologous sequences during repair and is considered a central player in preserving genome stability [14]. However, in the context of retroviral infection, RAD51's role becomes more complex. Some findings suggest that RAD51 may act as a barrier to integration by promoting the repair of viral DNA before integration. In contrast, others indicate a possible proviral function in facilitating post-integration repair or resolving replication stress [15, 16].

This review aims to comprehensively examine the role of the RAD51 recombinase in the life cycle, replication, and pathogenesis of human retroviruses. Specifically, the review seeks to elucidate how RAD51-mediated HR influences viral integration, genome stability, host-virus interactions, and the cellular DNA damage response (DDR). By synthesizing current evidence, this study aims to highlight RAD51 as a potential therapeutic target and to identify gaps in knowledge that warrant further investigation in the context of retroviral infections such as HIV-1 and HTLV-1.

## Homologous recombination

HR is a high-fidelity DNA repair pathway that plays a critical role in maintaining genomic stability. It is primarily responsible for the accurate repair of DSBs, which are among the most lethal forms of DNA damage [17, 18]. HR operates by using an undamaged homologous DNA sequence typically the sister chromatid as a template to guide the repair process, thereby ensuring precise restoration of the original DNA sequence [17].

Defects in the HR pathway have profound implications for cancer development. Mutations in genes encoding HR components, most notably *BRCA1* and *BRCA2*, are strongly associated with hereditary breast, ovarian, pancreatic, and prostate cancers [17–19]. These mutations impair the cell's ability to accurately repair DSBs, leading to the accumulation of genomic instability a hallmark of cancer. In such contexts, cells may rely on error-prone repair mechanisms like non-homologous end joining (NHEJ), further exacerbating mutational burdens [20, 21].

Conversely, some cancers exhibit elevated HR activity, which can enhance tumor cell survival under genotoxic stress, including chemotherapy and radiation [19, 22]. This dual role, where both HR deficiency and hyperactivation can contribute to tumorigenesis and therapy resistance, highlights the complex involvement of HR in cancer biology. As a result, the HR pathway has emerged as a promising target for cancer therapy, exemplified by the clinical success of PARP inhibitors in treating *BRCA*-mutant tumors through synthetic lethality [22].

**Fig. 1:**
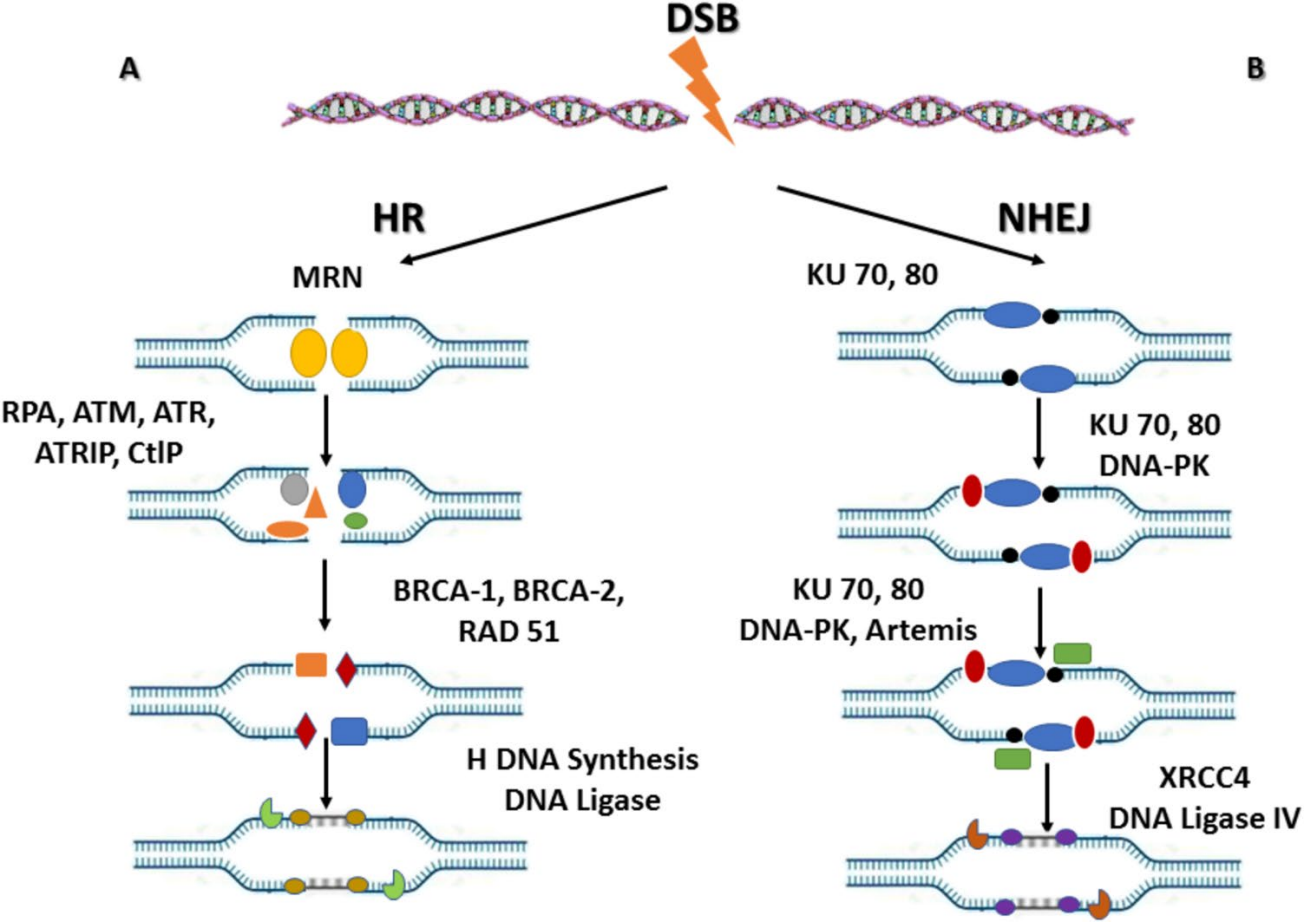
DNA Double-Strand Break (DSB) Repair Pathways. **A** Homologous Recombination (HR) Repair: The MRN complex composed of MRE11, RAD50, and NBS1 recognizes DNA double-strand breaks and activates ATM kinase to initiate the DNA damage response. End resection proceeds in the 5′ to 3′ direction via CtIP, producing single-stranded DNA (ssDNA) that is rapidly coated by replication protein A (RPA). ATR activation promotes HR progression. RAD51 subsequently replaces RPA on ssDNA to form the RAD51 nucleoprotein filament, which searches for a homologous sequence and initiates strand invasion. DNA synthesis, branch migration, ligation, and Holliday junction resolution restore the broken DNA with high fidelity. **B** Non-Homologous End Joining (NHEJ) Repair: In the NHEJ pathway, the DNA ends are directly bound and stabilized by the KU70/80 complex and DNA-PK. After end processing, final ligation is performed by XRCC4-Ligase IV, completing repair without requiring a homologous DNA template

Therefore, key proteins involved in HR include RAD51, BRCA1, BRCA2, and members of the MRN complex (MRE11-RAD50-NBS1), all of which coordinate the detection of DSBs, strand invasion, and the subsequent resolution of recombination intermediates [18, 19] (Fig. 1).

Understanding the mechanisms regulating HR and its dysregulation in cancer is essential for developing novel diagnostic markers and targeted therapies, particularly in the era of precision oncology.

Among the key regulators of HR, BRCA1 and BRCA2 play essential roles in coordinating end resection and in loading RAD51 onto single-stranded DNA (ssDNA) to form the active nucleoprotein filament required for strand invasion. Because RAD51 activity and its regulation by BRCA proteins directly influence genome stability and the cellular response to retroviral DNA intermediates, understanding BRCA1/BRCA2 function provides an important foundation for examining their involvement in retrovirus infection.

## The role of BRCA1 and BRCA2 in retrovirus infection

Because RAD51 activity is tightly controlled by BRCA (Breast Cancer)-family mediators, particularly BRCA2 which directly loads RAD51 onto single-stranded DNA, the next section examines how BRCA1/BRCA2 regulate RAD51 and why this regulation matters for retroviral infection and integration.

*BRCA1* and *BRCA2* are tumor suppressor genes that play critical roles in the maintenance of genomic stability through their involvement in the HR pathway of DNA repair [23]. These genes encode large, multifunctional proteins that act at several key stages of the DDR, particularly in the accurate repair of DSBs one of the most lethal forms of genomic damage [23].

BRCA1 functions primarily in the early steps of the HR pathway, where it facilitates DSB recognition, chromatin remodeling, and DNA end resection. It also plays a role in cell-cycle checkpoint activation and the coordination of the DNA repair machinery. BRCA2 acts downstream by directly regulating the RAD51 recombinase, promoting its loading onto ssDNA and stabilizing the RAD51 nucleoprotein filament, which is essential for strand invasion and homology search during HR [24].

Germline mutations in *BRCA1* or *BRCA2* dramatically increase the risk of developing several types of cancer, particularly breast, ovarian, prostate, and pancreatic cancers [23, 25, 26]. These mutations compromise HR efficiency, leading to genomic instability and increased reliance on alternative, error-prone DNA repair pathways. As a result, *BRCA*-deficient tumors are susceptible to DNA-damaging agents and poly (ADP-ribose) polymerase (PARP) inhibitors, which exploit the concept of synthetic lethality.

Beyond their clinical relevance as cancer predisposition genes, *BRCA1* and *BRCA2* have become central to our understanding of DNA repair biology, and their dysfunction represents both a diagnostic marker and a therapeutic vulnerability in precision oncology.

Zimmerman et al. [27] in their study, investigate the molecular mechanisms underlying G2 cell-cycle arrest induced by the HIV-1 viral protein R (Vpr). The authors demonstrate that Vpr-mediated G2 arrest requires the DNA damage checkpoint proteins Rad17 and Hus1, which are components of the ATR-dependent signaling pathway. Vpr expression also leads to the accumulation of DNA damage markers, including nuclear foci of *BRCA1* and *γ-H2AX*, suggesting activation of the DDR [27]. These findings support the model that Vpr induces a pseudo-DNA damage signal that hijacks the host cell's checkpoint machinery to block cell-cycle progression, potentially facilitating viral replication and persistence.

In Guendel et al. [28] found that their study uncovers a previously unrecognized role for the tumor suppressor protein BRCA1 in the context of HIV-1 infection. The authors demonstrate that BRCA1 acts as a transcriptional cofactor that enhances HIV-1 gene expression, particularly by cooperating with the viral transactivator Tat [28]. BRCA1 was shown to localize to the HIV-1 long terminal repeat (*LTR*) promoter region and facilitate Tat-mediated transactivation, suggesting it contributes to efficient viral transcription [28]. These findings reveal a novel function of BRCA1 beyond its canonical role in DNA repair and highlight a potential mechanism by which HIV-1 exploits host cellular machinery to support its replication.

Moreover, besides the HIV infection, the BRCA family may also be involved in HTLV pathogenesis. Shukrun et al. [29] examine how the HTLV-1 oncoprotein Tax disrupts the regulation of BRCA1 expression in their study. The authors show that under normal conditions, estrogen receptor alpha (ERα) activates *BRCA1* transcription through recruitment of the coactivators CBP/p300. However, the Tax protein interferes with this process by binding to CBP/p300, thereby preventing their association with ERα [29]. As a result, estrogen-induced BRCA1 expression is suppressed. These findings highlight a novel mechanism by which HTLV-1 Tax may contribute to genomic instability and tumorigenesis, particularly by impairing BRCA1-mediated DNA repair pathways through disruption of hormone signaling and transcriptional regulation.

In Jabareen et al. [30] in their study examined how 12-O-tetradecanoylphorbol-13-acetate (TPA), a known activator of protein kinase C, and the HTLV-1 Tax oncoprotein influence the expression of *BRCA1* and *estrogen response element (ERE)*-regulated genes. The authors demonstrate that both TPA and Tax modulate transcriptional activity of *ERE*-driven genes, with Tax notably suppressing *BRCA1* expression [30]. This suppression occurs through interference with the estrogen receptor signaling pathway, likely via competition for transcriptional coactivators such as CBP/p300 [30]. The findings suggest that HTLV-1 Tax, in synergy with TPA-induced signaling, can dysregulate hormone-responsive gene expression, potentially contributing to impaired DNA repair and oncogenic transformation in hormone-sensitive tissues.

Furthermore, Yaslianifard et al. [31] compared gene expression profiles between two distinct outcomes of HTLV-1 infection: HTLV-1-associated myelopathy/tropical spastic paraparesis (HAM/TSP) and ATLL. The authors report that *BRCA1* and *CHUK* (encoding IKK-α) are significantly upregulated in HAM/TSP. In contrast, genes involved in immune regulation, hormone signaling, and metabolism namely *NFKBIA*, *ESR1*, *PIK3R1*, and *PPARG* are downregulated compared to ATLL [31]. These contrasting expression patterns suggest divergent host responses and molecular pathways underlying the neurological versus oncogenic manifestations of HTLV-1 infection [31]. The findings highlight key regulatory genes that may contribute to the distinct pathophysiological mechanisms in HAM/TSP and ATLL, offering insights into disease progression and potential therapeutic targets.

In the context of viral infections such as HIV-1 and HTLV-1, studies reveal that HIV-1 exploits DDR mechanisms and co-opts BRCA1 to enhance viral transcription. At the same time, HTLV-1 oncoprotein Tax disrupts BRCA1 regulation by interfering with hormone receptor signaling and transcriptional coactivators, contributing to genomic instability and oncogenesis (see Table 1 for a concise summary of BRCA1-related studies in HIV-1 and HTLV-1). These findings deepen our understanding of virus-host interactions and point to the BRCA1 and associated signaling networks as possible critical mediators and potential therapeutic targets in virus-induced pathologies.

**Table 1:** Summarizes key studies that describe how BRCA1 participates in HIV-1 and HTLV-1 infection, highlighting its roles in DNA damage signaling, transcriptional regulation, and viral pathogenesis.

Study	Virus	Key molecule(s)	Main findings	Implications
Zimmerman et al. [27]	HIV-1	Vpr, BRCA1, Rad17, Hus1, γ-H2AX	Vpr induces G2 arrest via the ATR pathway; it activates DNA damage response (DDR) with BRCA1/γ-H2AX nuclear foci	Vpr mimics DNA damage to hijack the checkpoint, supporting viral replication
Guendel et al. [28]	HIV-1	BRCA1, Tat	BRCA1 enhances HIV-1 transcription by cooperating with Tat at the *LTR* promoter	BRCA1 has a non-canonical role in supporting viral gene expression
Shukrun et al. [29]	HTLV-1	Tax, BRCA1, ERα, CBP/p300	Tax inhibits ERα/CBP-p300-mediated BRCA1 transcription	HTLV-1 Tax disrupts BRCA1 regulation, promoting genomic instability
Jabareen et al. [30]	HTLV-1	Tax, BRCA1, ERα, CBP/p300, TPA	Tax and TPA suppress BRCA1 and ERE-driven genes via ERα signaling interference	Hormone-responsive gene dysregulation may contribute to oncogenesis
Yaslianifard et al. [31]	HTLV-1	BRCA1, CHUK, NFKBIA, ESR1, PIK3R1, PPARG	BRCA1 and CHUK are upregulated in HAM/TSP; other immune/hormone/metabolic genes are downregulated in ATLL	Gene expression profiles differentiate neurological (HAM/TSP) from oncogenic (ATLL) HTLV-1 outcomes

Notably, the report describing BRCA1 as a cofactor for HIV-1 Tat-mediated transcription is explicitly sourced [28], given its non-canonical nature.

## RAD51 and its function in retrovirus infection:

The HR is an error-free repair pathway in response to DNA-DSBs, which represent one of the most severe forms of genomic damage, that uses a homologous DNA sequence as a template [32]. A central player in this process is RAD51, a highly conserved recombinase protein critical for the homologous pairing and strand exchange steps of HR [32, 33].

**Fig. 2:**
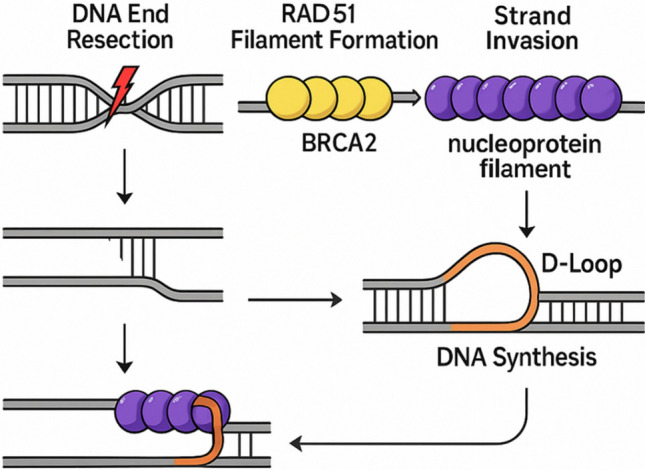
RAD51 filament formation and strand invasion during homologous recombination**.** Following DNA resection, RPA initially coats ssDNA; BRCA2 mediates displacement of RPA and loading of RAD51 onto ssDNA (callout). RAD51 forms a helical nucleoprotein filament that performs homology search and catalyzes strand invasion; the D-loop formed by the invading 3′ end is explicitly shown. *Schematic; not to scale*

Following DSB formation, the DNA ends are resected to generate ssDNA, which is rapidly coated by replication protein A (RPA) [34, 35]. RAD51 is then loaded onto the ssDNA with the help of several mediator proteins, including BRCA2, replacing RPA to form a nucleoprotein filament [34, 35]. This RAD51-ssDNA filament conducts a homology search in the sister chromatid or homologous chromosome and facilitates strand invasion into the homologous duplex DNA [34, 35]. This invasion forms a displacement loop (D-loop), enabling DNA synthesis using the undamaged template (Fig. 2).

RAD51's function is essential not only for faithful DNA repair but also for maintaining genomic integrity during replication and after DNA damage. Dysregulation of RAD51, whether through mutation or aberrant expression, is implicated in cancer development and resistance to therapy. Given its pivotal role in HR and genome maintenance, RAD51 is both a biomarker and a potential target for cancer therapy.

A previous study demonstrated that HIV uses the HR mechanism for its genome assembly or replication [36, 37]. Desfarges et al. [16] demonstrate that HIV-1 integrase (IN), expressed alone in *Saccharomyces cerevisiae*, is sufficient to catalyze chromosomal insertion of *LTR*-flanked DNA substrates with the characteristic 5-bp target site duplications, mirroring integration specificity seen in human cells. Critically, the study reveals that deletion of the yeast *RAD51* gene significantly increases integration by IN. At the same time, in vitro assays show that human RAD51 directly binds to integrase and inhibits concerted integration reactions [16]. These findings may identify RAD51 as a negative regulator of HIV-1 integration, acting through direct interaction with IN and potentially competing for substrate DNA binding, thus positioning RAD51 as a potential cellular restriction factor that modulates retroviral integration efficiency [16].

Chipitsyna et al. [38] demonstrated that the HIV-1 Tat protein enhances cell survival following treatment with the DNA-damaging agent cisplatin by upregulating RAD51, a key enzyme in HR-DNA repair. Tat-expressing cells show reduced DNA damage, fewer chromosomal abnormalities, and increased HR activity, while NHEJ components like Ku70 are slightly downregulated [38]. This shift from error-prone NHEJ to error-free HR suggests that Tat promotes genomic stability under genotoxic stress, potentially contributing to HIV-1 persistence and resistance to DNA-damaging agents.

In addition, two years later, Chipitsyna et al. [39] reveals that RAD51, a DNA repair protein, plays an unexpected role in regulating HIV-1 transcription in astrocytes by interacting with C/EBP transcription factors and NF-κB signaling. RAD51 enhances both basal and Tat-stimulated HIV-1-*LTR* promoter activity, and this effect requires intact NF-κB binding sites in the − 120 to − 80 region of the *LTR *[39]. Silencing either RAD51 or NF-κB p65 significantly reduces *LTR* activity. Additionally, Tat increases RAD51 expression and nuclear localization, reinforcing *LTR*-driven viral transcription. Thus, RAD51 cooperates with C/EBP/NF-κB to support efficient HIV-1 gene expression in brain-resident astrocytes, potentially promoting viral persistence in the central nervous system.

Moreover, Nakai-Murakami et al. [40] proved that expression of HIV-1 Vpr activates the ATM kinase and its downstream effectors including γ-H2AX and phosphorylated Chk2-beyond the previously described ATR-mediated G₂ arrest pathway. Strikingly, Vpr promotes nuclear foci formation of RAD51 and BRCA1, dissociates p53-RAD51 interactions in chromatin, and significantly increases HR repair activity in an I-SceI reporter system [40]. This HR enhancement is abolished by the ATM inhibitor KU55933, supporting that ATM activation by Vpr is essential for boosting recombination efficiency [40]. These findings could unveil a novel role for Vpr in modulating host DNA repair pathways, specifically enhancing HR via ATM activation, and suggest implications for viral integration, persistence, and HIV-associated tumorigenesis.

Rom et al. [41] investigated that HIV-1 infection of primary human microglia significantly induces RAD51 expression up to 12-fold by day 10 post-infection coinciding with active viral replication. Elevated RAD51 enhances transcription from the HIV-1 *LTR* promoter by interacting with the p65 subunit of NF-κB and promoting its binding to the κB motif within the 120 to 80 region of the *LTR *[41]. This functional cooperation between RAD51 and NF-κB p65 amplifies both basal and infection-driven *LTR* activity, suggesting that RAD51 acts not only in DNA repair but also as a positive regulator of HIV-1 gene expression in microglial cells, thereby potentially aiding viral persistence in the central nervous system.

In addition, Kaminski et al. [42] show that HIV-1 infection and treatments activating NF-κB (e.g., PMA/TSA) induce *RAD51* expression in microglia and peripheral blood mononuclear cells (PBMCs), and that RAD51 cooperates with the NF-κB p65 subunit to promote transcription from the HIV-1-*LTR*, particularly through the κB motif located between nucleotides 120 and 80. RAD51 physically associates with NF-κB p65, enhances its binding to the *LTR*, and nuclear localization of RAD51 is dependent on NF-κB signaling [42]. Inhibition of RAD51 activity or knockdown of RAD51 or p65 significantly diminishes *LTR*-driven transcription. It reduces HIV-1 replication in PBMCs by over 50%, indicating that RAD51 functions as a positive regulator of HIV-1 gene expression via the NF-κB pathway and may represent a novel target for antiviral strategies [43].

Furthermore, their other studies demonstrated that RAD51 expression is upregulated during HIV-1 infection and upon NF-κB pathway activation [44]. RAD51 physically interacts with the NF-κB p65 subunit and enhances its binding to the HIV-1-*LTR*, particularly at the κB enhancer region [44]. This cooperation between RAD51 and NF-κB significantly increases HIV-1 *LTR* activity in microglial cells and PBMCs. Silencing RAD51 or inhibiting its function results in a marked decrease in HIV-1 gene expression and replication, highlighting RAD51 as a critical co-regulator of NF-κB–mediated viral transcription and a potential target for therapeutic intervention in HIV-1 infection [43, 44].

In Cosnefroy et al. [15] revealed that formation of an active human RAD51 (hRAD51) nucleofilament is essential to inhibit HIV-1-IN activity both in vitro and in cells. Under conditions promoting hRAD51 polymerization on DNA, RAD51 destabilizes the integrase–DNA complex, thereby disrupting concerted integration of viral DNA into the host genome [15]. Moreover, chemical stimulation of endogenous RAD51 in HIV-1–infected cells enhances HR repair while simultaneously blocking viral integration, reducing infection efficiency [15]. These findings may identify RAD51 nucleofilament formation as a natural cellular barrier to HIV-1 integration, suggesting a novel therapeutic strategy to restrict retroviral infection by enhancing RAD51 activity.

Furthermore, Cosnefroy et al. [45] demonstrated that enhancing the formation of RAD51 nucleofilaments significantly inhibits HIV-1 integration both in vitro and in cell-based models. The study shows that RAD51 polymerizes on DNA and interferes with the formation and stability of the HIV-1 integrase–DNA complex, thereby preventing the concerted integration of viral DNA into host chromosomes [45]. Furthermore, pharmacological agents that stimulate RAD51 activity increase HR repair while simultaneously reducing HIV-1 infection efficiency [45]. These findings suggested that RAD51 acts as an intrinsic antiviral factor and that promoting RAD51 filament formation could represent a promising therapeutic approach to block HIV-1 integration and limit viral propagation.

In Panchapakesan et al. [46] offered the mechanistic explanation for how HIV-1 reverse transcriptase (RT) can generate non-homologous recombination events leading to sequence motif duplications. The authors propose that these duplications, widespread and variable in subtype C (e.g., PTAP motif duplications in p6-Gag), arise from template switches during reverse transcription that occur without perfect sequence homology [46]. By analyzing viral sequence databases, they classify motif duplications into four subgroups based on molecular context, and integrate this classification into an expanded model rooted in dynamic copy-choice and dock and lock paradigms to account for the observed length polymorphisms. Importantly, they suggest that some of these duplication events confer a replication fitness advantage, providing a selective benefit [46]. In aggregate, the study advances our understanding of how RT-mediated non-homologous template switching contributes to HIV-1 genome diversification and viral adaptation.

Moreover, RAD51 activation is observed in another human retrovirus, such as simian-human immunodeficiency virus (SHIV). Fujita et al. [47] described the successful construction of a novel SHIV (SHIV-97ZA012) by harnessing host-cell HR to insert the env gene from a subtype C clinical HIV-1 isolate into a SIV backbone. The researchers co-transfected lymphoid cells with overlapping PCR fragments of the SIV backbone and a 4 kb env fragment from the 97ZA012 isolate; intracellular recombination generated a full-length chimeric provirus, which was then serially passaged in rhesus macaque PBMCs [47]. The resulting SHIV-97ZA012 was replication-competent in macaque PBMCs and alveolar macrophages, used CCR5 as a co-receptor, and caused high plasma viremia along with transient but severe lung CD4⁺ T-cell depletion upon infection of rhesus macaques [47]. This data may demonstrate that intracellular HR can efficiently generate functional SHIVs encoding clinically relevant env genes, providing valuable models for HIV-1 pathogenesis and vaccine research.

In addition, Baydoun et al. [48] revealed that the HTLV-1 accessory protein p30 disrupts accurate DNA repair by inhibiting HR in favor of error-prone NHEJ. Mechanistically, p30 interferes with the formation of the MRE11-RAD50-NBS1 (MRN) complex, a critical initiator of HR, and dampens ATM activation, thereby preventing faithful repair via HR and shifting DNA double-strand break repair toward NHEJ pathways [48]. This bias toward mutagenic NHEJ allows accumulation of genomic instability in infected T cells, potentially contributing to oncogenic transformation in ATLL.

Furthermore, Ramezani et al. [49], by their cross-sectional study of PBMCs from 18 ATLL patients, 10 HAM/TSP cases, and 18 HTLV-1 asymptomatic carriers, measured proviral load (PVL) alongside expression of host genes *LAT*, *BIM*, *c-FOS*, and *RAD51* using qRT-PCR. ATLL patients exhibited significantly elevated PVL (~ 11,430 vs. ~ 530 copies per 10^4 cells, *p* < 0.001) [49]. While *BIM* and *c-FOS* levels were mildly higher in ATLL, only *RAD51* and *LAT* showed dramatic upregulation *RAD51* by ~ 160-fold and *LAT* by ~ 36-fold compared to carriers (both *p* < 0.001) [49]. Notably, *RAD51* expression positively correlated with HTLV-1 proviral load. These findings suggest that increased *RAD51* and *LAT* may reflect active viral replication and TCR signaling in ATLL, although *RAD51* upregulation alone does not suffice to prevent DNA damage during malignant transformation.

Based on the mentioned studies, RAD51 emerges as a multifaceted player at the intersection of DNA repair, viral replication, and transcriptional regulation in the context of HIV-1 and HTLV-1 infections. In HIV-1-infected cells, RAD51 expression is upregulated by viral proteins such as Tat and Vpr, enhancing HR and contributing to genomic stability, viral persistence, and increased transcription from the viral *LTR* via cooperation with NF-κB. Conversely, stimulation of RAD51 nucleofilament formation can restrict HIV-1 integration, highlighting its dual role as both a facilitator and a potential restriction factor. This observation positions RAD51 as a restriction factor during early HIV-1 infection [15, 16].

In HTLV-1 infection, however, viral proteins like p30 inhibit RAD51-mediated HR, promoting unfaithful DNA repair and genomic instability a hallmark of oncogenic transformation in ATLL. Elevated RAD51 expression in ATLL further underscores its involvement in disease progression, though it appears insufficient to counteract the DNA damage induced by viral interference. Together, these findings suggest that RAD51 is co-opted differently by retroviruses: as a transcriptional coactivator and repair modulator in HIV-1, and as a dysregulated repair factor in HTLV-1-associated oncogenesis, offering insights into potential therapeutic strategies targeting RAD51's activity in viral infection and malignancy.

Collectively, in HIV-1, RAD51 is upregulated and can support *LTR* transcription, whereas in HTLV-1, p30 suppresses HR and shifts double-strand break repair toward the non-homologous end-joining pathway.

Table 2 provides a consolidated overview of published studies examining RAD51 in HIV-1, SHIV, and HTLV-1 infection, illustrating its dual functions as both a proviral co-factor and an antiviral restriction element.

**Table 2:** The role of RAD51 in HIV-1, SHIV, and HTLV-1 Studies

Study	Virus	RAD51 role/effect	Main findings
HIV-1 Tat increases cell survival in response to cisplatin by stimulating Rad51 gene expression	HIV-1	Upregulated by Tat	Enhances cell survival by promoting homologous recombination (HR) repair of DNA damage
Cooperativity between Rad51 and C/EBP family transcription factors modulates basal and Tat-induced activation of the HIV-1 LTR in astrocytes	HIV-1	Transcriptional coactivator	RAD51 enhances Tat-induced HIV-1 *LTR* activity through interaction with C/EBP transcription factors
Chromosomal integration of LTR-flanked DNA in yeast expressing HIV-1 integrase: downregulation by RAD51	HIV-1	Restriction factor	RAD51 overexpression inhibits integration of *LTR*-flanked DNA by HIV-1 integrase
HIV-1 Vpr induces an ATM-dependent cellular signal with enhanced homologous recombination	HIV-1	HR stimulator	Vpr activates the ATM pathway, leading to upregulation of RAD51 and enhanced HR
Activation of HIV-1 LTR by Rad51 in microglial cells	HIV-1	Transcriptional activator	RAD51 directly activates HIV-1 *LTR* in microglia, supporting viral gene expression
Stimulation of the human RAD51 nucleofilament restricts HIV-1 integration in vitro and infected cells	HIV-1	Restriction factor	Pre-assembled RAD51 nucleofilaments inhibit HIV-1 integration, acting as a barrier to infection
Interplay of Rad51 with NF-κB Pathway Stimulates Expression of HIV-1	HIV-1	Transcriptional co-regulator	RAD51 interacts with NF-κB to promote HIV-1 *LTR* activity and viral expression in immune cells
A Model of Non-Homologous Recombination Mediated by HIV-1 Reverse Transcriptase Explaining Sequence Motif Duplications That Confer a Replication Fitness Advantage	HIV-1	Not directly involved	Describes reverse transcriptase-mediated non-HR; RAD51 is not central to the mechanism
Human T-lymphotropic type 1 virus p30 inhibits homologous recombination and favors unfaithful DNA repair	HTLV-1	HR inhibitor	p30 inhibits RAD51-mediated HR, shifts repair toward error-prone pathways, promoting genomic instability
Assessment of HTLV-1 proviral load, LAT, BIM, c-FOS, and RAD51 gene expression in adult T-cell leukemia/lymphoma	HTLV-1	Overexpressed	RAD51 is significantly upregulated in ATLL patients and correlates with high proviral load

## Therapeutic approach based on HR pathway

Most therapeutic strategies targeting the HR pathway in the context of retroviral infection are supported primarily by preclinical evidence, with clinical applications currently established mainly in HR-deficient and BRCA-mutated cancers. PARP inhibitors have emerged as a transformative class of targeted cancer therapeutics, particularly effective in tumors with deficiencies in the HR-DNA repair pathway [50]. PARP enzymes are essential for detecting and repairing single-strand DNA breaks via the base excision repair pathway [50, 51].

**Fig. 3:**
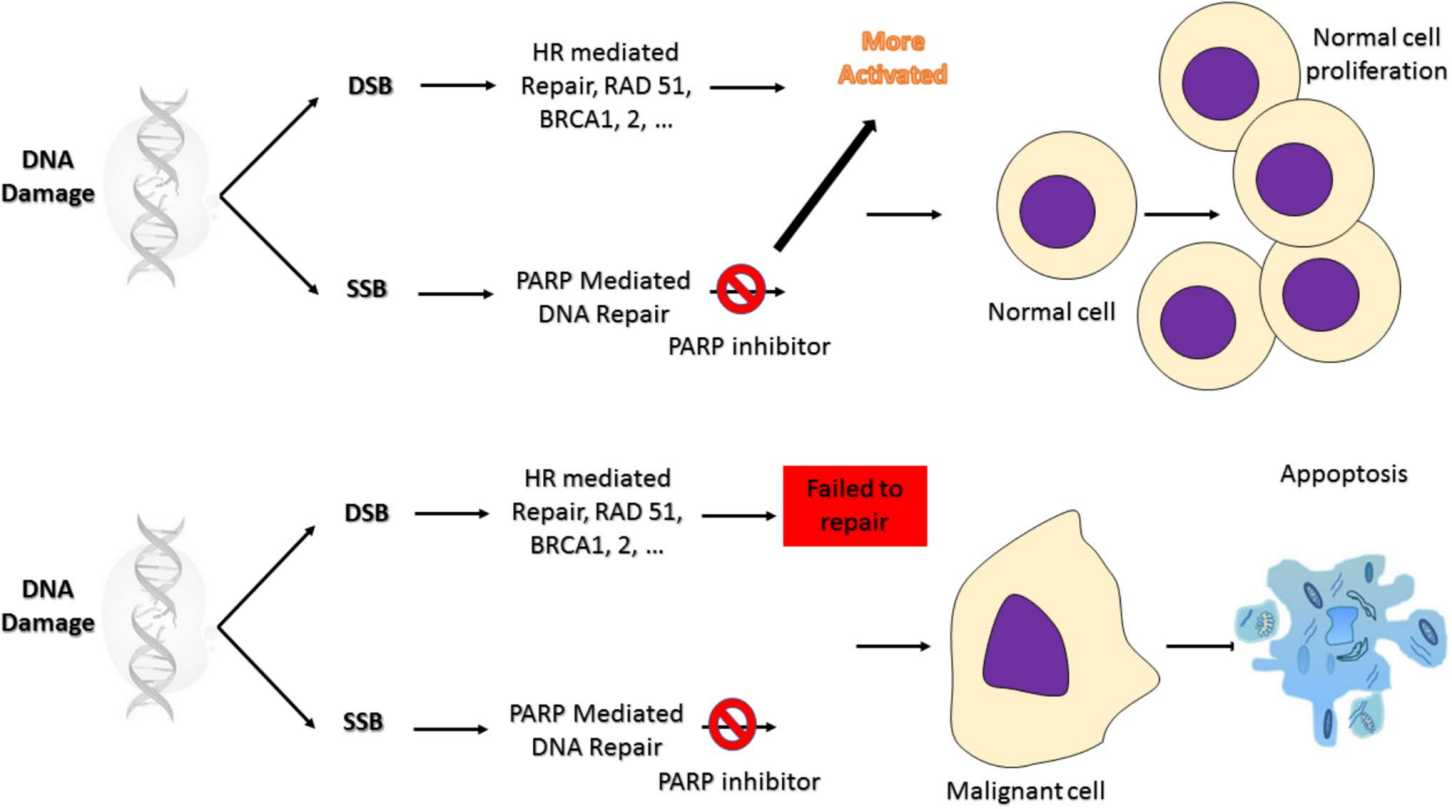
Mechanism of PARP inhibitors in HR-deficient cancer cells. This diagram illustrates the therapeutic rationale behind the use of PARP inhibitors in tumors with homologous recombination (HR) deficiencies, such as those harboring *BRCA1* or *BRCA2* mutations. In normal cells, PARP enzymes detect and repair single-strand breaks (SSBs) through the base excision repair pathway, maintaining genomic integrity. Administration of a PARP inhibitor blocks this repair mechanism, leading to persistent SSBs. In HR-deficient cells, these unrepaired SSBs are converted into double-strand breaks (DSBs) during DNA replication. Because HR-deficient cells lack the machinery to accurately repair DSBs, the accumulation of damage leads to collapsed replication forks and activation of error-prone repair pathways. This event ultimately causes genomic instability and cell death a phenomenon termed synthetic lethality

The preclinical studies demonstrated that, in HR-deficient cells such as those harboring mutations in *BRCA1* or *BRCA2,* PARP inhibition leads to the accumulation of unrepaired single-strand breaks that collapse into DSBs during DNA replication [50, 51]. Because these cells lack functional HR to repair such damage accurately, they become highly reliant on error-prone repair pathways, ultimately resulting in genomic instability and cell death a concept known as synthetic lethality (Fig. 3).

This mechanism has been clinically exploited in treating *BRCA*-mutant breast, ovarian, prostate, and pancreatic cancers, and ongoing research aims to extend PARP inhibitor use to broader patient populations with HR deficiencies or replication stress signatures [50, 51]. Thus, PARP inhibitors could represent a powerful example of precision oncology, targeting specific vulnerabilities in tumor DNA repair machinery.

### PARP inhibitor in human retrovirus infection

Tibebe et al. [52] assessed the potential of combining a histone deacetylase (HDAC) inhibitor (vorinostat) with olaparib (PARP inhibitor), rucaparib (PARP inhibitor), niraparib (PARP inhibitor), or talazoparib (PARP inhibitor) to enhance HIV latency reversal. While PARP inhibitors alone show no latency-reversing activity, when used in concert with vorinostat, they increase viral reactivation roughly threefold over HDAC inhibitors alone in J-Lat and primary CD4⁺ T-cell models. Mechanistically, this synergy is linked to inhibition of tankyrase, a PARP family enzyme that modulates Hippo signaling and influences BRD4 recruitment to the HIV-1 *LTR* promoter [52]. The combination therapy also achieved a 67% reduction in reservoir size in primary infected cells. These results provide evidence that dual inhibition targeting both chromatin remodeling and PARP-mediated signaling can significantly improve latency reversal and reduce the reservoir, supporting further development of this combined "kick-and-kill” [52].

The therapeutic effect of chemical and synthetic drugs by direct or indirect targeting of PARP function is being further investigated. Anderson et al. [53], in their study, uncovered that PARP1 functions as a transcriptional coactivator of the HTLV-1 oncoprotein Tax, enhancing Tax-mediated activation of the viral *LTR*. In purified in vitro transcription assays, basal *LTR* transcription supported by general transcription factors was not responsive to Tax alone, but inclusion of PARP1 enabled robust Tax-dependent activation. Additionally, in cellular systems, PARP1 significantly augmented Tax-induced *LTR*-driven transcription, evidencing its essential role as a coactivator in both biochemical and in vivo context [53]. In addition, Ishitsuka et al. revealed that arsenic trioxide (As₂O₃) effectively induces apoptosis in HTLV-1 infected T-cell lines (MT-1, MT-2) and fresh ATLL patient cells at clinically relevant doses (1–2 μM), through activation of caspase 3, 8, and 9, loss of mitochondrial membrane potential, and cleavage of PARP-despite no involvement of CD95/Fas or TNF-α receptor signaling and resistance to death receptor pathway blockade using receptor-neutralizing antibodies [54].

Moreover, Machijima et al. [55] demonstrated that indole-3-carbinol (I3C), a bioactive compound found in cruciferous vegetables, exerts selective antitumor activity against HTLV-1 infected T-cell lines and ATLL patient cells, without inhibiting normal T cells or uninfected lines. I3C induces dose-dependent G₁/S cell-cycle arrest by downregulating cyclin D1, D2, Cdk4, and Cdk6. It triggers apoptosis through reduced expression of anti-apoptotic proteins (XIAP, survivin, Bcl-2), increased *Bak* expression, and activation of caspase 3, 8, 9, and PARP cleavage. It is important to note that PARP cleavage represents a marker of caspase activation and apoptosis, whereas PARP inhibitors act through enzymatic PARP inhibition and synthetic lethality rather than through PARP cleavage. Mechanistically, it suppresses NF-κB and AP-1 signaling via reduced IκBα phosphorylation and JunD expression. In a mouse xenograft model, oral I3C (50 mg/kg/day) significantly inhibited ATLL tumor growth with tolerable toxicity. These findings support I3C as a promising, host-tolerated natural therapeutic agent for HTLV-1 associated ATL [55].

Deguelin, a naturally derived rotenoid, potently inhibits the proliferation of HTLV-1-transformed T-cell lines (KUT-1 and MT-2) by inducing dose and time-dependent growth arrest and apoptosis (IC₅₀ ≈ 0.5 µM) [56]. Mechanistically, it suppresses constitutive phosphorylation of STAT3 and downregulates the anti-apoptotic protein survivin, leading to activation of caspases 3, 8, and 9, cleavage of PARP, and loss of mitochondrial potential. The apoptotic effects are proteasome-dependent: treatment with MG132 reverses deguelin-induced STAT3 dephosphorylation and survivin degradation, suggesting proteasomal destruction of phosphorylated STAT3 and survivin as part of the mechanism [56]. These findings position deguelin as a promising therapeutic candidate against ATLL, particularly in cases refractory to conventional treatments.

Zimmerman et al. [57], investigated the orally bioavailable histone deacetylase inhibitor AR-42 and demonstrated potent antitumor activity against HTLV-1–associated ATLL. AR-42 induced histone H3 hyperacetylation, triggered dose-dependent apoptosis via cytochrome c release and PARP cleavage, and suppressed the growth of ATL-derived cell lines (MT-2, C8166) [57]. In a NOD/SCID mouse model engrafted with ATL cells, dietary AR-42 significantly reduced tumor burden as indicated by decreased serum IL-2Rα levels and extended survival compared to untreated controls [57]. These results support further development of AR-42 as a promising HDAC inhibitor therapeutic for refractory HTLV-1 driven lymphoid malignancies.

In Suzuki et al. [58] demonstrated that Resveratrol exerts potent anti-proliferative effects on HTLV-1infected T-cell lines (MT-2 and HUT-102) by inducing caspase-dependent apoptosis through inhibition of STAT3 phosphorylation at Tyr705 and Ser727, as well as downregulation of anti-apoptotic proteins Mcl-1 and cIAP-2. Treated cells display activated caspase three cleavage and PARP cleavage, confirming engagement of intrinsic apoptotic pathways. Similar cytotoxic effects are observed with a STAT3-specific inhibitor (S3I-201), spotlighting STAT3 as a critical therapeutic target in ATLL [58]. Collectively, these findings suggest that Resveratrol holds promise as a natural therapeutic agent for ATL via the disruption of STAT3-dependent survival signaling.

Bai et al. [59] demonstrate that the small-molecule PARP inhibitor PJ-34 exerts potent anti-proliferative and pro-apoptotic effects against HTLV-1–transformed and ATLL patient-derived cell lines. PJ-34 treatment induces G2/M cell-cycle arrest, reactivates p53 signaling (including increased p21, BAX, MDM2, and GADD45 expression), and triggers caspase 3-dependent mitochondrial apoptosis even in p53-null cells [59]. The compound also results in the accumulation of DNA damage and loss of mitochondrial membrane potential in susceptible cells. While most ATLL cell lines are PJ-34 sensitive, resistance observed in specific lines (MT-2, C91PL) is associated with failure to activate caspase-3 and elevated *RelA/p65* expression [59]. Overall, this work positions PJ-34 as a promising therapeutic agent for ATLL, especially in cases exhibiting defects in HR-DNA repair and limited treatment options.

In this year, Sasaki et al. [60], by their study, declared that the small-molecule survivin suppressant YM155 significantly inhibits proliferation and induces apoptosis in sensitive lines (S1T and MT-1) via downregulation of survivin, activation of caspase-3, and PARP cleavage [60]. Resistance in MT-2 cells is linked to constitutive activation of STAT3, STAT5, and AKT; however, combining YM155 with the STAT3 inhibitor S3I-201 overcomes resistance and significantly suppresses proliferation in these cells [60]. These findings support YM155’s potential as a novel therapeutic candidate for ATL, particularly in cases lacking durable treatment response due to survivin overexpression or STAT3-driven drug tolerance.

In Houssein et al. [61] demonstrated that combining thymoquinone (TQ) with As₂O₃ and interferon-α (As/IFN-α) significantly enhances anti-leukemic activity in HTLV-1 infected T-cell lines and in a mouse xenograft model of ATLL. While the standard As/IFN-α regimen alone induced apoptosis and growth arrest in HTLV-1-positive cells, the addition of TQ markedly sensitized both HTLV-1-positive and negative T-cell lines, leading to greater inhibition of viability and more pronounced induction of apoptosis evident through mitochondrial membrane depolarization, caspase-3 and PARP cleavage, and shifts in apoptotic regulators (downregulated *Bcl-2* and *XIAP*; upregulated *Bax*) [61]. In the HuT-102 xenograft model, the triple combination significantly reduced tumor volume compared to single or double-agent treatments, suggesting that TQ enhances the therapeutic efficacy of As/IFN-α while potentially allowing dose reduction of arsenic to mitigate toxicity [61].

Dimethyl fumarate (DMF) exhibits potent antitumor activity in HTLV-1 infected ATLL cell lines (MT-1 and MT-2) by simultaneously suppressing both canonical and non-canonical NF-κB signaling and inhibiting constitutive STAT3 phosphorylation [62]. This dual blockade leads to reduced expression of anti-apoptotic proteins c-IAP2 and survivin, culminating in induction of caspase-dependent apoptosis and impaired cell proliferation [62]. These findings support DMF as a promising novel therapeutic candidate for ATL, acting through coordinated disruption of survival pathways central to HTLV-1–mediated oncogenesis.

Kato et al. [63], demonstrates that the selective CDK9 inhibitor LY2857785 (also known as BAY 1143572) exerts vigorous antitumor activity against HTLV-1 associated ATLL both in vitro and in vivo. Treatment of ATL-derived cell lines and primary patient cells led to reduced phosphorylation of RNA polymerase II Ser2, downregulated expression of anti-apoptotic proteins (such as MCL-1 and c-Myc), and induction of caspase-3 mediated apoptosis, including PARP cleavage [63]. Remarkably, LY2857785 also triggered autophagy, as evidenced by increased LC3-II formation. In NOD/SCID mice bearing ATL xenografts, LY2857785 significantly inhibited tumor growth and prolonged survival.

Collectively, these studies highlight a promising spectrum of targeted therapeutic strategies for HIV and HTLV-1 infection, especially in adult T-cell leukemia lymphoma. Agents such as PARP inhibitors (PJ-34), survivin suppressants (YM155), and natural compounds including Resveratrol, thymoquinone, indole-3-carbinol, deguelin, and DMF have demonstrated the ability to disrupt key survival and proliferation pathways namely NF-κB, STAT3, and anti-apoptotic regulators such as survivin, Mcl-1, and cIAP-2. Furthermore, emerging epigenetic and transcriptional therapies, such as HDAC inhibitors (AR-42) and CDK9 inhibitors, effectively induce apoptosis, autophagy, and tumor regression in preclinical ATL models. These findings underscore the therapeutic value of simultaneously targeting viral oncogene signaling, host DNA repair defects, and transcriptional dependencies. They also support a paradigm shift toward combination or synthetic lethality-based therapies to overcome resistance and improve clinical outcomes in patients with ATL and other HTLV-1 associated malignancies. Table 3 summarizes major therapeutic approaches targeting DNA repair, transcriptional regulation, and survival pathways in HIV and HTLV-1-associated disease, including agents under investigation for latency reversal and ATLL.

**Table 3:** Therapeutic Interventions in HIV/HTLV-1-associated latency and leukemia

Study	Agent(s)	Mechanism	Main findings
Tibebe et al. [52]	Vorinostat + PARP inhibitors	HDAC inhibition + tankyrase/PARP signaling inhibition	Enhanced HIV latency reversal (~ 3x); 67% reservoir reduction
Anderson et al. [53]	PARP1	Acts as a Tax coactivator	PARP1 enhances HTLV-1 Tax-induced transcription
Ishitsuka et al. [54]	Arsenic trioxide	Mitochondrial apoptosis independent of death receptors	Effective apoptosis in HTLV-1-infected cells, PARP cleavage
Machijima et al. [55]	Indole-3-carbinol (I3C)	NF-κB/AP-1 inhibition, G1/S arrest	Selective cytotoxicity, apoptosis induction, and tumor growth inhibition
Ito et al. [56]	Deguelin	STAT3 and survivin suppression	Caspase activation, mitochondrial dysfunction, proteasome dependence
Zimmerman et al. [57]	AR-42 (HDACi)	Histone acetylation, cytochrome c release	Tumor growth suppression, extended survival in mice
Suzuki et al. [58]	Resveratrol	STAT3 inhibition, downregulates Mcl-1, cIAP2	Caspase activation, apoptosis in HTLV-1 cells
Bai et al. [59]	PJ-34 (PARPi)	G2/M arrest, p53 signaling	Apoptosis via caspase-3 is effective in most ATLL lines
Sasaki et al. [60]	YM155	Survivin suppression	Induces apoptosis; STAT3 inhibition restores sensitivity
Houssein et al. [61]	TQ + As/IFN-α	Mitochondrial depolarization, Bax/Bcl-2 shift	Enhanced tumor suppression and apoptosis in xenografts
Maeta et al. [62]	Dimethyl fumarate	NF-κB and STAT3 inhibition	Reduces survivin, cIAP2; induces apoptosis
Kato et al. [63]	LY2857785 (CDK9i)	Inhibits RNA Pol II; c-Myc/MCL-1 downregulation	Induces apoptosis and autophagy; suppresses tumor growth

## Conclusion and future directions

RAD51 emerges as a central, yet context-dependent, player at the intersection of DNA repair and retroviral infection. In HIV-1, it supports viral persistence by enhancing HR and transcriptional activation, notably through cooperation with viral proteins and NF-κB signaling. In contrast, HTLV-1 impairs RAD51-mediated repair to favor genomic instability and tumorigenesis, particularly in ATLL. These opposing mechanisms demonstrate how RAD51 can function as both a viral cofactor and a restriction element, depending on the viral strategy. Future investigations should focus on the direct molecular interfaces between RAD51 and retroviral proteins, the role of RAD51 in HIV latency and reactivation, and its potential as a therapeutic target either by enhancing its antiviral properties or by exploiting synthetic lethality in virus-associated cancers. As in both retroviral infections, the expression of RAD51 is increased as a biomarker associated with viral persistence. Pharmacological modulation of RAD51, particularly in combination with PARP inhibitors or transcriptional regulators, holds promise for novel therapeutic strategies against both HIV-1 persistence and HTLV-1 driven oncogenesis.

Future research targeting RAD51's interaction with viral components and its regulation may uncover novel therapeutic opportunities for limiting retroviral propagation and virus-induced malignancies. Future studies can be included in the investigation of the direct molecular interfaces between RAD51 and key retroviral proteins such as HIV-1 Tat, Vpr, and HTLV-1 p30. Structural and biochemical studies (e.g., co-crystallization, crosslinking mass spectrometry) could uncover how these interactions modulate RAD51's recombinase activity or its recruitment to DNA damage sites.

In addition, targeting RAD51 as an antiviral strategy by pharmacological inhibition of RAD51 (or its nucleofilament formation) could serve as a novel antiviral approach in HIV infection. These warrants testing of RAD51 inhibitors in HIV-1–infected primary cells and animal models, especially in synergy with existing antiretroviral therapies.

Moreover, the role of RAD51 in Latent HIV-1 Reactivation should be considered since RAD51 interacts with NF-κB and enhances HIV-1 *LTR* activation; its role in latent reservoir reactivation should be assessed. Does RAD51 contribute to HIV latency reversal under certain stimuli? This has implications for "shock-and-kill" cure strategies. In HTLV-1-induced oncogenesis, despite RAD51 being upregulated in ATLL, it does not seem to prevent genomic instability. Future work should explore whether this upregulation reflects a compensatory but ineffective response to DNA damage, or whether RAD51 overexpression paradoxically supports survival of genetically unstable cells.

Finally, the therapeutic modulation of RAD51 with small molecules or synthetic lethality approaches (e.g., in combination with PARP inhibitors), such as Amuvatinib, should be explored as a potential strategy to treat Virus-Associated Cancers since HR activity (and RAD51) is often elevated in virus-related malignancies (e.g., ATLL) (64, 65).

## Data Availability

Not applicable (no new datasets generated or analyzed in this review).
